# Distinct Microbial Limitations in Litter and Underlying Soil Revealed by Carbon and Nutrient Fertilization in a Tropical Rainforest

**DOI:** 10.1371/journal.pone.0049990

**Published:** 2012-12-13

**Authors:** Nicolas Fanin, Sandra Barantal, Nathalie Fromin, Heidy Schimann, Patrick Schevin, Stephan Hättenschwiler

**Affiliations:** 1 Centre d'Ecologie Fonctionnelle et Evolutive (CEFE), CNRS, Montpellier, France; 2 Université of Montpellier II, Montpellier, France; 3 UMR Ecologie des Forêts de Guyane (EcoFoG), Campus Agronomique, Kourou, French Guiana; Utrecht University, The Netherlands

## Abstract

Human-caused alterations of the carbon and nutrient cycles are expected to impact tropical ecosystems in the near future. Here we evaluated how a combined change in carbon (C), nitrogen (N) and phosphorus (P) availability affects soil and litter microbial respiration and litter decomposition in an undisturbed Amazonian rainforest in French Guiana. In a fully factorial C (as cellulose), N (as urea), and P (as phosphate) fertilization experiment we analyzed a total of 540 litterbag-soil pairs after a 158-day exposure in the field. Rates of substrate-induced respiration (SIR) measured in litter and litter mass loss were similarly affected by fertilization showing the strongest stimulation when N and P were added simultaneously. The stimulating NP effect on litter SIR increased considerably with increasing initial dissolved organic carbon (DOC) concentrations in litter, suggesting that the combined availability of N, P, and a labile C source has a particularly strong effect on microbial activity. Cellulose fertilization, however, did not further stimulate the NP effect. In contrast to litter SIR and litter mass loss, soil SIR was reduced with N fertilization and showed only a positive effect in response to P fertilization that was further enhanced with additional C fertilization. Our data suggest that increased nutrient enrichment in the studied Amazonian rainforest can considerably change microbial activity and litter decomposition, and that these effects differ between the litter layer and the underlying soil. Any resulting change in relative C and nutrient fluxes between the litter layer and the soil can have important consequences for biogeochemical cycles in tropical forest ecosystems.

## Introduction

Over the last two decades, considerable efforts were made towards a better understanding of the effects of global change factors such as climate change or nutrient deposition on the quality of plant litter, its subsequent decomposition and the consequences on ecosystem carbon (C) dynamics [1]–[4]. Whether or not C is sequestered in forest ecosystems depends on the often small difference between photosynthetic C fixation and ecosystem respiration, with soil respiration representing between half to two thirds of the total ecosystem respiration [5]–[8]. Nutrient availability is a key factor in the regulation of soil respiration, and anthropogenic alterations of the nitrogen (N) and phosphorus (P) cycles can have important consequences for the net CO_2_ exchange between the biosphere and the atmosphere, and thus for the global C budget [9], [10].

Tropical forests are a particularly important component in the terrestrial C budget and even small changes in tropical CO_2_ fluxes may modify the global C cycle [11]–[14]. With a share of about 55% to 76% of total soil CO_2_ efflux from tropical soils [6], [15], [16] microbial-driven heterotrophic soil respiration is a critical CO_2_ flux to the atmosphere in tropical ecosystems. Microbial heterotrophs in the litter layer and the underlying soil are highly responsive to altered nutrient availability (*e.g.* [17]–[20]). N and P inputs in particular modify the soil C∶N∶P stoichiometry and that of plant residues, which in turn affect decomposer activity and growth [21], [22], and the processes of litter decomposition and organic matter mineralization [23], [24]. In addition to nutrient deposition, global change-induced shifts in plant tissue C quality (*e.g.* secondary metabolites, non-structural carbohydrates) [25]–[27] may also affect heterotrophic soil organisms. Such C-quality changes may have important consequences in some tropical forests where the poor C quality of leaf litter has been proposed to impose energy starvation on decomposers [28].

External resource supply through fertilization provides a straightforward experimental test of ecosystem nutrient limitation and the response of ecosystem processes to altered resource availability [10], [29], [30]. Despite a large diversity in geology, soil characteristics, climatic factors and biological diversity of tropical rainforests [31], [32], only a relatively small number of fertilization experiments have been performed in this biome. Some of these experiments reported positive effects of P fertilization on decomposition and on CO_2_ release into the atmosphere [33]–[36], while others have found an increase of soil C stocks associated with lower soil respiration following N additions [37]–[39], or contrasting effects of these resources as a result of site age related soil fertility gradients [40]–[42]. Additionally, studies that included a combination of different resources in their fertilization protocol observed interacting effects between resources such as N×P on litter mass loss or microbial activity during decomposition [41]–[43], suggesting that some limiting resources can influence the fate and the impact of other resources on soil processes [29], [30]. However, only few studies have simultaneously manipulated the availability of all three key elements C, N, and P and none of them were performed in a lowland tropical forest. By adding a labile C source (*i.e.* cane sugar, glucose monohydrate) in factorial CNP fertilization designs, important and significant interactions between C×P or C×N on microbial responses and soil C dynamics have been reported in a tropical montane forest of Ecuador [44] and along a successional gradient in a temperate system [45]. Although these studies provide clear evidence of interactions between C and nutrients, sugars used as C fertilization may produce immediate responses by favoring opportunistic soil organisms. Other, more complex, but still relatively easily accessible C sources - such as cellulose - should be tested to provide a more detailed understanding of the potential regulation of soil processes by multiple elements.

The distinction between microbial processes in the litter layer and the underlying soil are rarely made clearly [46], [47], and the effects of resource addition on litter and soil heterotrophs are seldom addressed in the same study [17]. Although the litter layer and the underlying soil are intimately connected through the exchange of energy and matter, microclimatic and physical conditions as well as the chemical composition, with notably a stark contrast in organic matter quality and C∶N∶P stoichiometry, differ strongly. Leaf litter material display much wider C to nutrient ratios as well as distinct C quality compared to that of soil organic matter, with especially more labile compounds in freshly fallen leaf litter. These different qualities of organic substrates available for microbial heterotrophs might result in distinct constraints for litter and soil communities. In addition, the very high tree species richness, typical for most tropical forests, results in chemically diverse leaf litter inputs at small spatial scales [48], [49]. These distinct litter substrates decompose at different rates [50], [51] and affect the respiration and structure of microbial communities in the underlying soil differently [52], [53]. It is important to account for this chemical heterogeneity of tropical leaf litter when assessing the effects of increased resource supply, because nutrient enrichment effects are likely to be modified by differences in initial litter quality. In fact, in a companion paper, Barantal and coll. [43] demonstrated that combined fertilization with N and P increasingly stimulated leaf litter decomposition with decreasing initial litter P concentration and increasing initial litter N∶P ratios. Moreover, these positive NP fertilization effects were enhanced when soil fauna had access to decomposing litter [43]. It is widely accepted that soil fauna are important decomposers in tropical rainforests [51], [54]–[57], and McGlynn and coll. [58] showed that soil C∶P stoichiometry controls soil fauna abundance in a Costa Rican rainforest. However, the importance of litter identity in the response of decomposition and associated microbial processes (especially in the underlying soil) to fertilization, as well as the role of fauna in modulating this response, remains little explored.

In this study, we addressed the question of how multiple resource fertilizations affect heterotrophic processes within decomposing leaf litter and in the underlying soil, and investigated how these effects are influenced by species differences in litter quality and the presence of soil fauna. Data were collected from an ongoing fertilization experiment in a low-fertile lowland rainforest in French Guiana where C (cellulose), N (urea) and P (phosphate) are added in a fully factorial fertilization experiment since 2009 [43]. We specifically addressed the following hypotheses: (i) external supply of readily available C, N and P alleviates resource limitation and consequently stimulates the overall microbial capacity (estimated by substrate induced respiration, SIR); (ii) the stimulating effects of external resource supply increase with decreasing initial litter quality; (iii) the previously reported fauna-induced stimulation of fertilization effects on decomposition [43] translates into increased consumption of microorganisms by litter-feeding fauna and consequently decreases microbial respiration; (iv) the response of SIR to external resources differ between litter and the underlying soil, with stronger effects of nutrient addition on litter SIR because soil organic matter has a lower C to nutrient ratio compared to leaf litter, and stronger effects of C fertilization on soil microbial respiration compared to litter because litter is richer in labile C substrates than soil.

## Materials and Methods

All necessary permits were obtained for the described field studies (fertilization), and no specific permits were required for the described measurements in the field (sampling of soil and leaf litter), in agreement with the owner, the French research center CIRAD. We confirm that the field studies did not involve endangered or protected species.

### Study site

The study site is located within the undisturbed Amazonian rainforest of Paracou near Sinnamary, French Guiana (5°15′N, 53°′W). The mean annual air temperature is 25.5°C (10-year average, 1995–2005) with only slight intra annual variations. Total annual rainfall is approximately 2575 mm (10-year average, 1995–2005), with two distinct rainy seasons (a moderate one from December to February and a stronger one from April to July) with an associated range in relative air humidity between 70 and 90% [59]. Tree species richness is around 150 species per hectare with a mean density of 620 individual trees ha^−1^ (individuals of a diameter >0.1 m at breast height) [60]. Soils in the study area are classified as acrisol, developed over a Precambrian metamorphic formation called the Bonidoro series. The soil is nutrient-poor with 24% clay, 7% silt and 69% sand, and a pH (water extract) of 4.7 in the top 0.2 m [57]. Average soil C∶N is 14.7 with a total C of 2.21 g kg^−1^, a total N of 0.15 g kg^−1^ soil and a total P of 0.010 g kg^−1^ soil (for more details on soil composition and texture see [53]).

### Plant material

For the construction of litterbags we used leaf litter from the six tree species *Carapa procera* (Aublet), *Goupia glabra* (Aublet), *Platonia insignis* (Martius), *Hymenaea courbaril* (Linnaeus), *Simarouba amara* (Aublet) and *Vochysia tomentosa* (G. Mey.) (Table 1). A representative pool of fresh fallen leaf litter of each species was obtained from a tree plantation close to our study site. These more than 25-year-old tree stands have been established using local seed sources and have a fully closed canopy composed of about 40 individuals of each of a total of 16 tree species growing in monocultures [61]. Litter was collected twice a month during the year 2009 in suspended 25 m^2^ litter traps and pooled across sampling dates. Leaves with obvious signs of damage (*e.g.* herbivory, galls, fungal attacks) and green leaves were excluded (typically <15% of total collected leaves). Leaf litter was air-dried, weighed (8.0±0.1 g air-dry to oven-dry corrected mass per litterbag) and enclosed in plastic mesh bags for each species individually. We used coarse-mesh (8 mm) and fine-mesh (0.06 mm) bags in order to allow or not the access of soil and litter macrofauna. The initial quality of pooled leaf litter differed significantly among the six species (Table 1). For example, the C∶N ratio varied between 34.5 (*P. insignis*) and 51.5 (*C. procera*), and the N∶P ratio varied between 21.8 (*H. courbaril*) and 78.9 (*P. insignis*).

**Table 1:** Initial litter quality parameters measured for leaf litter from the six different tree species used in our study.

Litter characteristics^†^	*C. procera*	*G. glabra*	*H. courbaril*	*P. insignis*	*S. amara*	*V. tomentosa*
Litter elements (%DM)						
Carbon	48.4±0.2	49.7±0.2	49.7±0.1	49.0±0.2	49.1±0.1	42.9±0.4
Nitrogen	0.94±0.04	1.21±0.13	1.22±0.03	1.42±0.03	1.11±0.07	0.87±0.04
Phosphorus	0.019±0.012	0.033±0.004	0.056±0.002	0.018±0.001	0.032±0.002	0.029±0.001
Litter stoichiometry						
C∶N	51.5±2.1	41.1±4.1	40.7±1.1	34.5±0.6	44.2±2.9	49.3±2.9
C∶P	2547±147	1507±168	888±36	2722±154	1534±111	1479±87
N∶P	49.5±2.9	36.7±1.5	21.8±0.4	78.9±5.6	34.7±0.7	30±2.9
Carbon compounds (%DM)						
Dissolved organic carbon	0.59±0.09	1.93±0.24	0.56±0.02	1.46±0.16	1.07±0.07	0.74±0.03
Water soluble compounds	32.4±0.3	36.6±0.4	31.0±1.0	29.3±0.3	45.4±0.4	34.6±1.1
Hemicellulose	7.5±0.5	16.2±0.7	10.3±0.1	23.5±0.7	11.7±0.2	20.1±1.1
Cellulose	22.7±0.4	18.8±0.3	22.3±0.6	22.5±0.7	20.0±0.3	19.7±0.4
Lignin	37.5±0.5	28.4±0.8	36.3±0.7	24.7±1.1	22.8±0.7	25.6±0.4
Soluble phenolics	2.8±0.2	1.1±0.2	1.0±0.1	1.0±0.1	4.4±0.2	0.6±0.1
Total phenolics	7.9±0.8	2.8±0.3	4.2±0.4	12.5±0.5	11.0±0.8	4.4±0.4
Condensed tannin	7.7±0.7	0.6±0.1	3.8±0.4	0.4±0.1	6.3±0.3	3.9±0.3

### Experimental design

A full-factorial fertilization experiment (control, C, N, P, CN, CP, NP, CNP) plus one additional fertilization treatment (called *+*other nut. throughout the paper) with major cations (K, Ca, Mg) and micronutrients (i.e. B, Cu, Fe, Mn, Mo, S, Zn) was set up in the field using a total of five blocks. Each of the five blocks measured approximately 3000 m^2^ and was situated within a 2.5 ha zone of rather homogeneous flat topography. Each of the nine treatment plots within blocks measured 5.5 m×5.5 m and was separated from neighbor plots by a buffer zone of at least 5 m. Fertilization was applied twice a year during the two dry periods in order to limit potential wash-off of fertilizer just after application. The fertilization was started in April 2009 and is ongoing since then. Based on preliminary microcosm tests of different fertilizer concentrations (Barantal, *unpublished data*) and the concentrations used in other tropical fertilization experiments [34], [36], [41], we used the annual doses of 1405 kg C ha^−1^ year^−1^ provided as cellulose (Waterspare, celliob industry, France), 130 kg N ha^−1^ year^−1^ as coated urea [(NH_2_)_2_CO] and 69 kg P ha^−1^ year^−1^ as monopotassium phosphate [KH_2_PO_4_] corresponding to C∶N of 10.8, C∶P of 20.4 and N∶P of 1.9. The cations and micronutrients in the +other nut. treatment was equivalent to 22 kg ha^−1^ year^−1^ of a mixture of H_3_BO_3_ (1150 ppm), CuSO_4_ (1150 ppm), Fe-EDTA (2%), MnSO_4_ (1150 ppm), ZnSO_4_ (600 ppm) and (NH_4_)_2_MoO_4_ (600 ppm), plus 87 kg K ha^−1^ year^−1^ as K_2_SO_4_ , 92 kg Mg ha^−1^ year^−1^ as MgSO_4_, and 50 kg Ca ha^−1^ year^−1^as Ca-EDTA. Twelve 15 cm×15 cm large litterbags (6 litter species×2 mesh sizes) were randomly placed directly on the soil surface (natural litter was removed prior to litterbag placement), fixed on the forest floor with wire and exposed in each of the 45 plots for a total of five months from September 2009 (just before the second fertilization event) to February 2010.

### Sample collection

After 158 days of exposure in the field, the litterbags were retrieved and the underlying soil underneath each litterbag was collected, resulting in a total of 540 pairs of litterbag-soil samples (5 blocks×9 treatments×6 species×2 mesh size). The underlying soil was sampled in the center of the litterbag using a stainless steel cylinder (diameter of 5 cm) to a depth of 8 cm. All sampling was done from 9^th^ to 14^th^ February 2010 during the wet season, approximately two months after peak litter fall [59]. In the laboratory, litter from the litterbags was weighed for total fresh mass and an aliquot (2 g fresh weight) was dried at 65°C to determine litter dry mass and litter mass loss. The remaining litter material of each litterbag was air-dried and stored dry until further analyses. Soil samples were air-dried, passed through a 2 mm sieve to remove roots and stones, homogenized and stored dry until further analyses.

### Determination of soil and litter SIR

Substrate induced respiration, SIR, as a measurement of potential activity, encompasses several aspects of the microbial community, and is often used as a proxy of the soil respiration process [62]. It was used as an indicator of the overall capacity of the litter and soil microbial communities [53]. Soil SIR was measured according to Beare and coll. [63]. For each sample, 10 g of soil (dry weight) were placed in a sealed plasma flask of 150 ml. A solution of glucose (1.5 mg C g^−1^ of dry soil) was added to reach 80% of field capacity. The flasks were incubated at 25°C for 6 h, a time span that is considered short enough to avoid *de novo* enzyme synthesis. Two hundred µl air samples from the headspace of each flask were analyzed for CO_2_ concentration after 2 and 6 h incubation with a gas chromatograph using a microcatharometer (VARIAN GC 4900; Varian, Walnut Creek, USA). From the amount of CO_2_ released during this time we calculated SIR expressed in µg of C-CO_2_ per g of soil per hour. Litter SIR was measured in the same way with the exception that we used 2 g of litter material (dry weight) and 2 ml of a solution of glucose to supply 20 mg C g^−1^ of dry litter mass. For some of the most rapidly decomposing litter types (19% of all litter samples collected in the field) there was not enough litter material left for these measurements, but each combination (litter species×fertilization treatment) was replicated at least three times, allowing robust statistical analyses.

### Data analysis

Normality of the distribution of data was assessed for all variables using Shapiro-Wilkinson's test and the homogeneity of variance using the Fisher (*F*) test. When data were not normally distributed, transformations of variables were performed in order to meet the assumptions before any further statistical tests. In particular, litter mass loss, litter SIR and soil SIR rates were log-transformed.

The effect of species and mesh size on litter mass loss, litter SIR and soil SIR without fertilization was assessed with linear mixed models, LMM, in control plots only (using the “nlme” R package [64], [65]). Blocks were considered as a random factor while litter species, mesh size and their interactions were fixed factors. To evaluate the relationship in control plots between litter species-specific initial quality and litter mass loss, litter SIR and soil SIR, we performed stepwise regression to select the best litter quality predictor when soil fauna were included or not. We divided the data into two sets based on mesh size before running the statistical analysis. The results of stepwise regression should be interpreted with caution because this method leads to several biases such as errors in parameter estimation, inconsistencies among model selection algorithms or reliance on a single best model [66].

We analyzed the effect of fertilization in two steps. First, to test for the effect of any of the major resources C, N or P added, the effect of fertilization was assessed with full factorial LMM (for these tests the *+*other nut. treatment was excluded in order to keep a balanced design). In these analyses, we compared all plots receiving C-, N- or P- fertilization and all plots with no addition of this particular resource (C, N or P presence/absence in each combination). Blocks were considered as random factor while C, N, P supply, litter species, mesh size and their interactions were fixed factors. Second, to test for the effect of each external resource singly or in combination with each other, a “net fertilization effect” was calculated within each block as the difference for response variables between the plot receiving a given fertilization treatment and the control plot. A positive net fertilization effect denoted higher mass loss or SIR with fertilization. When significant effects were found, we ran post-hoc means separation tests using Tukey-HSD (α = 0.05).

Mathematical correlations between litter mass loss and litter and soil SIR rates were explored with simple linear or non-linear regressions. Regression analyses were also used to assess potential relationships between the “net fertilization effect” and initial litter quality in order to evaluate whether the effect size depended on specific initial litter quality traits. Levels of significance are indicated as * (*p*<0.05), ** (*p*<0.001), and *** (*p*<0.0001). All statistical tests were performed with the R software (version 2.11.1).

## Results

### Litter mass loss and SIR without fertilization

In the unfertilized control plots we observed a mesh size effect on litter mass loss, but not on litter SIR and soil SIR (Table 2). Fauna access to litterbags increased mass loss, and this effect depended on litter species identity (significant species×mesh size interaction, Table 2). Fauna access also led to variation in litter mass loss (from 30.3% in *C. procera* to 68.3% in *G. glabra*), and to a higher variation (CV = 30%) compared to small mesh width litterbags (range between 23.5% in *V. tomentosa* and 37.2% in *H. courbaril* with a CV of 12%, Table 3, Table S1).

**Table 2:** Results of mixed linear models to test for the effects of litterbag mesh size and litter species identity on (a) litter mass loss, (b) SIR litter and (c) soil SIR within control plots only (no fertilization).

(a) Litter mass loss	Num. d.f.†	Den. d.f.†	*F value*	*p-value*
mesh size	1	43	**19.1**	**<0.0001**
species	5	43	**5.9**	**0.0003**
mesh size × species	5	43	**4.1**	**0.004**

(b) SIR litter	Num. d.f.	Den. d.f.	*F value*	*p-value*
mesh size	1	34	2.0	0.17
species	5	34	**14.7**	**<0.0001**
mesh size × species	5	34	1.3	0.28

(c) SIR soil	Num. d.f.	Den. d.f.	*F value*	*p-value*
mesh size	1	44	0.6	0.43
species	5	44	**2.9**	**0.02**
mesh size × species	5	44	1.5	0.20

† Num d.f., numerator degrees of freedom; Den d.f., denominator degrees of freedom.

**Table 3:** Means (± SE) and CV (in %) of litter mass loss, litter SIR, and soil SIR measured in control plots (no fertilization) with or without fauna access.

Variable	Mean	CV	Best Predictor	Effect	*r^2^*	*p-value*
**With fauna access**						
Litter mass loss	44.2±13.9	30	Dissolved Organic Carbon	+	**0.89**	**0.005**
			Condensed Tannins	−	**0.74**	**0.02**
SIR litter	18.2±3.9	23	Dissolved Organic Carbon	+	0.58	0.07
SIR soil	1.44±0.72	47	Lignin	−	0.59	0.07
**Without fauna**						
Litter mass loss	31.6±3.9	12	Total Carbon	+	**0.76**	**0.02**
SIR litter	19.5±4.3	21	Total Carbon	+	0.49	0.12
SIR soil	1.33±0.76	59	Dissolved Organic Carbon	+	**0.69**	**0.04**

When several litter quality traits significantly explained litter mass loss or SIR, all the corresponding models from stepwise regression analysis are displayed (in bold). When no litter trait significantly explained the variable (*p*>0.05), the best model is shown.

Similar to litter mass loss, litter SIR and soil SIR were also significantly different among litter species. Litter SIR was highest in decomposing *S. amara* litter (23.3 µg g^−1^ h^−1^) and lowest in *V. tomentosa* litter (12.9 µg g^−1^ h^−1^). In contrast, soil SIR was highest underneath *G. glabra* litter (1.83 µg g^−1^ h^−1^) and lowest underneath *H. courbaril* litter (1.03 µg g^−1^ h^−1^). However, in contrast to litter mass loss, litter SIR and soil SIR showed no significant mesh size×litter species interaction (Table 2).

The observed litter species effects on litter mass loss and SIR were related to initial litter carbon quality (Table 1, 3). The best predictor for litter mass loss when fauna had access to litterbags was the initial concentration of dissolved organic carbon (DOC) in leaf litter with increasing mass loss when DOC concentrations increased (r^2^ = 0.89, *p* = 0.05). In contrast, the concentration of condensed tannins (CT) showed a negative correlation with litter mass loss when fauna was present (r^2^ = 0.74, *p* = 0.02). Similar to litter mass loss, litter SIR showed a trend for a positive correlation with initial litter DOC in the presence of fauna (r^2^ = 0.58, *p* = 0.07), while soil SIR tended to correlate negatively with initial litter lignin content (r^2^ = 0.59, *p* = 0.07). Without macrofauna, litter mass loss correlated best and positively with initial concentrations of total carbon (r^2^ = 0.76, *p = *0.02) (Table 3). The same trend was found for litter SIR, while soil SIR without fauna access to litterbags was best predicted with the initial litter DOC concentration (increasing soil SIR with increasing DOC, r^2^ = 0.69, *p* = 0.04).

### Fertilization effects

**Figure 1:**
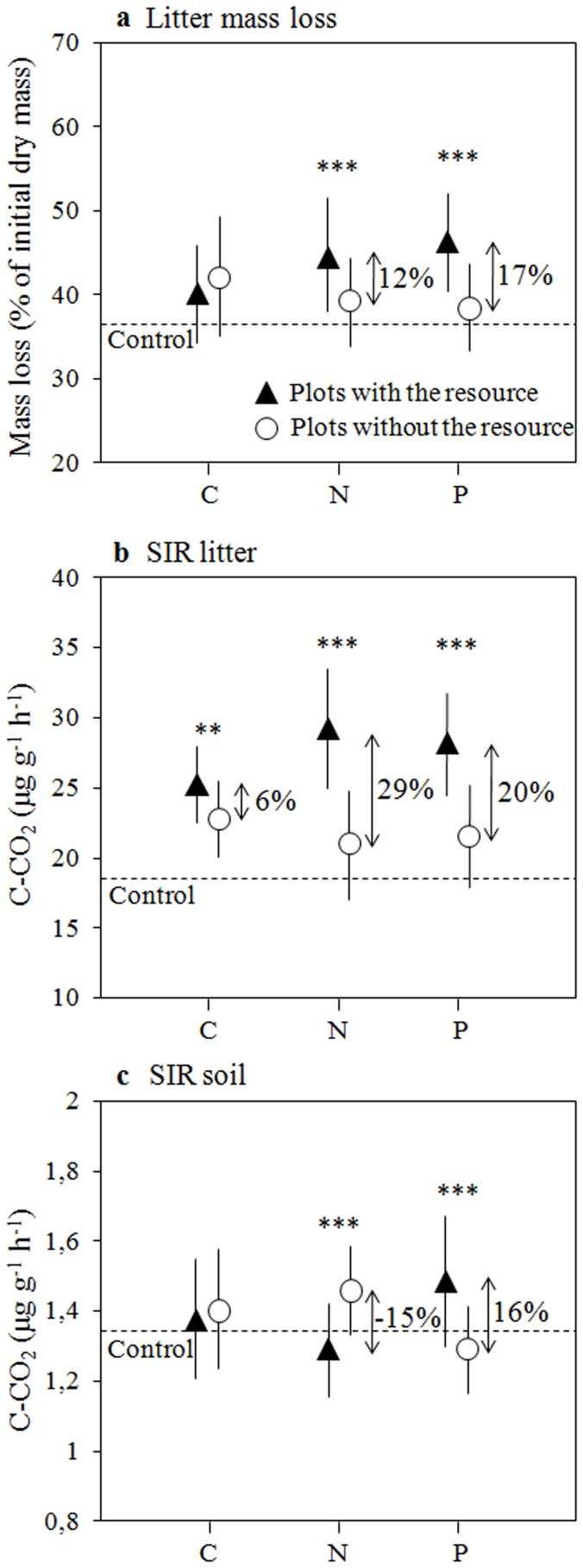
Effects of C, N, and P fertilization (alone or in any combination with the other resources) on (a) litter mass loss, (b) litter SIR and (c) soil SIR, without distinction of litter species and mesh size. These effects were analyzed using linear mixed models (dashed lines indicate the mean values of control plots). Black triangles represent the mean values (± SE) for all plots receiving C, N or P fertilization, and open circles the values for all plots receiving no addition of C, N or P, respectively (*e.g.* C, CN, CP and CNP vs control, N, P and NP for the C resource). Stars denote significant differences between plots with or without the addition of C, N or P as follows: * (*p*<0.05), ** (*p*<0.01), *** (*p*<0.001).

**Figure 2:**
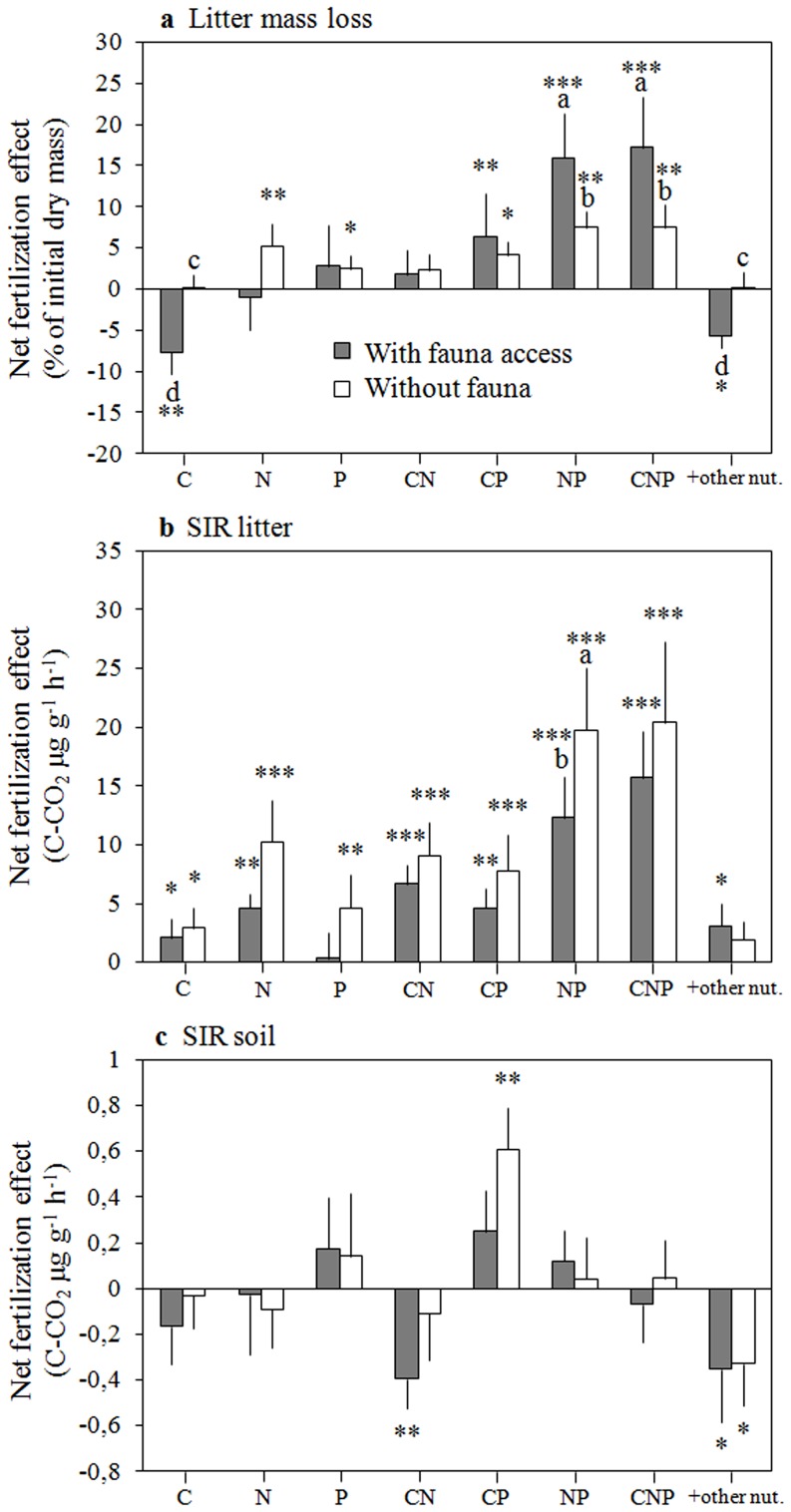
Net fertilization effects (mean ± SE) on (a) litter mass loss, (b) litter SIR and (c) soil SIR. Net fertilization effects are defined as the absolute difference between values measured on control plots and those measured on the plots of the respective fertilization treatment. Gray bars represent treatments with fauna access (coarse mesh litterbags) and open bars represent treatments without fauna access (fine mesh litterbags). Different letters indicate significant differences between coarse and fine mesh litterbags for a given treatment. Stars denote net treatment effects that are significantly different from zero using paired Student's *t* tests: * (*p*<0.05), ** (*p*<0.001), *** (*p*<0.0001).

In a first analysis of fertilization effects we identified how absolute litter mass loss and rates of litter and soil SIR differed with C, N, and P amendment compared to when these respective fertilizers were not added (*e.g*. all plots receiving C compared to all plots without C addition). The significant effects of mesh size and litter species identity on litter mass loss reported in control plots persisted in fertilized plots and explained a higher amount of variation in mass loss compared to that of C, N and P supply (Table 4). On average, N and P fertilization increased litter mass loss by 17% and by 12%, respectively (Figure 1). In contrast, C fertilization showed no significant effect on litter mass loss with a trend for negative effects (Figure 1, 2, Table 4). The effects of N and P fertilization both depended on litter species (significant N and P×litter species interactions, Table 4). The P fertilization was further influenced by mesh size with a stronger effect when fauna had access (significant P×mesh size interaction).

**Table 4:** Results from mixed linear models to test for the effects of fertilization (addition or not of either one of C, N, and P), litterbag mesh size, litter species identity, and their interactions on (a) litter mass loss, (b) litter SIR and (c) soil SIR.

(a) Litter mass loss	Num. d.f.†	Den. d.f.†	*F value*	*p-value*
C (Carbon)	1	436	1.7	0.19
N (Nitrogen)	1	436	35.5	<0.0001
P (Phosphorus)	1	436	47.6	<0.0001
mesh size	1	436	87.3	<0.0001
species	5	436	69.8	<0.0001
mesh size × species	5	436	26.6	<0.0001
P × mesh size	1	436	5.2	0.023
N × species	5	436	2.6	0.026
P × species	5	436	4.1	0.0012

(b) SIR litter	Num. d.f.	Den. d.f.	*F value*	*p-value*
C	1	358	13.6	0.0003
N	1	358	195.1	<0.0001
P	1	358	64.2	<0.0001
mesh size	1	358	24.4	<0.0001
species	5	358	62.6	<0.0001
C×N	1	358	4.89	0.028
N×P	1	358	4.5	0.034
mesh size × species	5	358	4.9	0.0002
P × species	5	358	3.6	0.0037
C × mesh size	1	358	5.3	0.022

(c) SIR soil	Num. d.f.	Den. d.f.	*F value*	*p-value*
C	1	461	1.1	0.30
N	1	461	21.0	<0.0001
P	1	461	20.9	<0.0001
mesh size	1	461	0.01	0.93
species	5	461	1.6	0.17
N×P	1	461	5.0	0.026

Only significant interaction terms are shown.

† Num d.f., numerator degrees of freedom; Den d.f., denominator degrees of freedom.

The litter species-specific differences in litter SIR remained essentially the same across fertilization treatments as those observed in control plots. However the mesh size effect was significant when fertilized plots were included in the analysis (Table 4). With fauna access, litter SIR was on average 17% lower than that measured in litter without fauna access. N and P fertilization explained a higher amount of variation in litter SIR than mesh size and litter species identity (Table 4). Overall, N and P fertilization increased litter SIR by 29% and by 20%, respectively (Figure 1). Carbon fertilization also significantly increased litter SIR by 6%. The positive C effect, however, was influenced by mesh size with a weaker C fertilization effect when fauna had access to the litter. Moreover, a positive interaction was observed between C and N addition and N and P addition (higher litter SIR when C or P was added with N simultaneously).

The significant litter species effect on soil SIR observed in control plots (Table 2) disappeared with fertilization, and mesh size still had no significant effect on soil SIR (Table 4). While the addition of N and P and their interaction significantly changed soil SIR, C addition had no impact (Figure 1, Table 4). On average, N fertilization decreased soil SIR by 15%. In contrast, P fertilization showed an average increase of 16% (Figure 1). Moreover, a negative interaction between N and P additions was observed (on average lower SIR than P alone when N was added in combination to P).

### Treatment specific net fertilization effects

In a second analysis we explored in more detail how litter mass loss and rates of litter and soil SIR changed in the eight different fertilization treatments compared to the control treatment (net fertilization effect = absolute difference between treatment and control). Litter mass loss and litter SIR both showed the highest net fertilization effects with a combined addition of N and P supply (Figure 2). These net NP fertilization effects were highest for mass loss when fauna had access (on average 35% higher than in control plots), and highest for litter SIR when fauna was excluded (on average 96% higher than in control plots). We observed broadly similar patterns for the net effects of the different fertilizer combinations on litter mass loss and litter SIR (Figure 2). However, the net fertilization effects on litter SIR were stronger than those on litter mass loss. Also, the presence of fauna tended to decrease litter SIR, and to rather increase litter mass loss, respectively. As a result, the overall positive relationship between litter SIR and litter mass loss across all fertilization treatments when fauna was absent disappeared in the presence of fauna (Figure S1).

Soil SIR responded distinctly to fertilization compared to litter SIR or litter mass loss (Figure 2). Most fertilization treatments showed no significant net effect on soil SIR rates, notably the combined addition of N and P that induced the strongest response on litter mass loss and litter SIR. The combined C and P fertilization was the only treatment showing a positive net effect on soil SIR when fauna was excluded from the litterbags (on average 46% higher than in control plots). This CP fertilization effect, however, was not statistically significant when fauna had access to the litterbags on top of the sampled soil. The net fertilization effects on soil SIR were negative when plots were either fertilized with CN or with cations and micronutrients (Figure 2). Soil SIR showed no correlation with litter SIR or litter mass loss.

### Litter species-specific responses to fertilization

**Figure 3:**
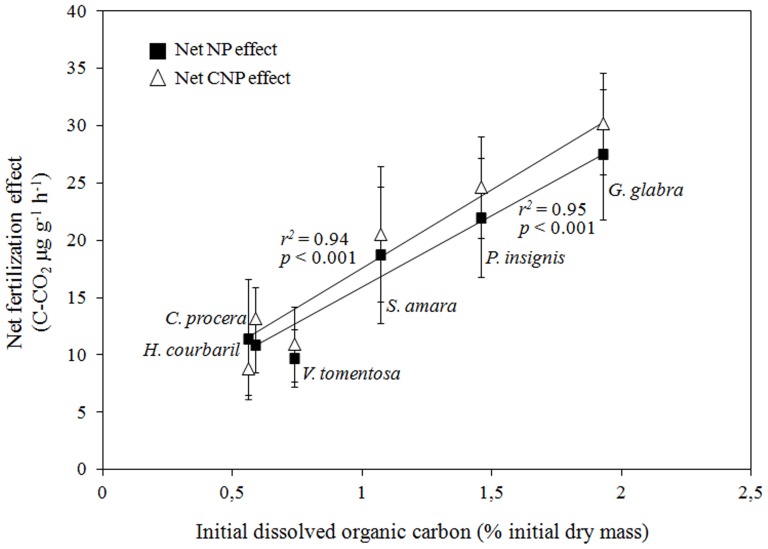
Net effects of NP (black squares) and CNP (open triangles) fertilization (mean ± SE) on litter SIR (data pooled across mesh-size) as a function of the initial litter species-specific DOC concentration. Net fertilization effects are defined as the absolute difference between values measured on control plots and those measured on the plots of the respective fertilization treatment.

The effects of nutrient fertilization on litter mass loss and litter SIR differed among litter species (significant nutrient×litter species interactions, Table 4) apparently as a result of distinct initial litter quality. For example, N fertilization effects increased with decreasing litter initial N concentration. Likewise, the P fertilization effect was particularly strong in litter of low initial P concentrations (*e.g*. *P. insignis*). Interestingly though, N and P fertilization effects on litter SIR were strongest in litter species with the highest initial DOC concentrations, *i.e.* in *G. glabra* (+37.9% litter SIR) and *S. amara* (+30.7% litter SIR) for N addition and in *P. insignis* (+34.2% litter SIR) and *G. glabra* (+26.2% litter SIR) for P addition. The strong effect of combined N and P fertilization observed for litter mass loss (Figure 2) depended on initial litter P concentrations, *i.e.* the net NP fertilization effect increased with decreasing litter P concentration. In contrast, the NP fertilization effect on litter SIR correlated best with initial concentrations of DOC, *i.e.* the net NP fertilization effect increased with increasing litter DOC concentration (Figure 3). This relationship was positive independently of the mesh-size. Additional fertilization with cellulose did not change these relationships between initial litter quality and the net NP fertilization effects.

## Discussion

### Decomposition and litter SIR in response to fertilization

In our first hypothesis we stated that in the studied low fertile Amazonian rainforest, an increased availability of the key resources C, N and P should increase leaf litter SIR in parallel to faster decomposition. We tested this hypothesis with a fully factorial fertilization experiment that was the first to our knowledge to use cellulose addition, a less labile C form than the commonly used highly labile sugars. In support of our hypothesis we found that litter mass loss and litter SIR both increased on P- and N-fertilized plots compared to plots that were not amended with P or N (Figure 1). Contrary to the predicted strong effect of P fertilization at our study site of particularly low soil P availability, our results suggest that both P and N limit litter microbial decomposers and decomposition simultaneously. In fact, the net fertilization effect of a combined P and N addition was clearly stronger than the effects of separate P or N fertilization, especially for SIR. The microbial communities in decomposing litter respired on average 85% more with a combined N and P supply compared to an increase of 11% and 31% with P and N supplied singly, respectively. Except for a small positive effect on litter SIR, C fertilization showed little effects. Litter mass loss was actually slower in plots fertilized with just C compared to control plots. Barantal and coll. [43] previously argued that decomposers might prefer cellulose to leaf litter that contains large quantities of recalcitrant C compounds. Such cellulose preference may explain the somewhat higher litter SIR and slower litter decomposition with cellulose fertilization. However, we can not exclude the possibility that this potential initial cellulose preference followed by enhanced litter SIR may lead to an increased consumption of litter C *via* a priming effect in the longer term [67], [68].

Our results are in line with those from previous studies in montane forests of Hawai'i showing that N and P together can constrain litter mass loss and microbial functioning during decomposition [41], [42], and support the increasing evidence that ecosystem processes are more often than not co-limited by N and P [10], [69]. Mineralization and acquisition of N from decomposing leaf litter material requires the breakdown of the C skeleton of rather complex organic compounds [70], [71]. In contrast, P is less strongly bound and may be lost from decomposing leaf litter at higher rates than N [72]. In addition, more than half of the total litter P may be readily available mineral phosphate in contrast to organic N that dominates the total litter N pool [49]. Therefore, P is more easily accessible than N in the early stages of litter decomposition, and N may initially be relatively more limiting. The relative importance of N availability should shift with increasing age and decreasing C∶N ratios of organic matter. Accordingly, N fertilization is expected to have less of an effect within the soil than P, which is in line with the observed positive P and a negative N effect on soil SIR in our study. A second, not mutually exclusive, explanation is that bacterial and fungal communities differ in their resource limitation. Indeed, in experimental manipulations of C (as glucose), N and P in a tropical montane rainforest in southern Ecuador, Krashevska and coll. [44] showed that fungi predominantly responded to N whereas bacteria responded to P. Consequently, such differences in primary limitation between fungi and bacteria may also explain the particularly strong effect on microbial functioning in the litter with a combined addition of N and P that stimulates both fungal and bacterial communities.

### Litter species-specific resource limitation

Large variation in green leaf quality and stoichiometry was observed among tropical tree species at regional scales [73], and a similar large variation in litter quality and stoichiometry has also been documented at small local scales [48], [50]. Such high interspecific variation in litter quality results in a spatially highly variable organic matter input to the soil, creating a mosaic of diverse resources for heterotrophic microbial communities [52], [53]. This variability in chemical quality of tree leaf litter was taken into account here by selecting litter from six tree species with contrasting stoichiometry and C quality. According to our second hypothesis we expected the relative effect of external resource supply to increase with decreasing initial litter quality.

In the litter layer, N and P fertilization interacted with litter species identity, suggesting that the response to nutrient addition were dependent on initial litter quality (Table 3). With a combined N and P fertilization, litter mass loss correlated negatively with initial litter P concentration and with decreasing initial litter N∶P ratios. Such relationship was also observed across various mixtures of the studied six litter species [43]. This influence of initial litter P status suggests a pivotal role of litter P availability in determining the strength of NP fertilization effects on litter decomposition. On the other hand, litter SIR correlated positively with initial litter DOC concentration under a combined N and P fertilization, indicating that microbial activity increased more with NP fertilization in litter with more labile C substrates (Figure 3). In a different NP fertilization experiment in a Costa Rican rainforest, the NP effect was strongest at the beginning of the wet season, when labile C content was maximal in litter leachates [34]. The results from the Costa Rican study and our own study both suggest that labile C compounds in leaf litter provide the microorganisms with the required energy to efficiently use external nutrients. Since the DOC from the litter used in our study should be available, particularly at the beginning of litter decay, the persistent interactive effect with fertilization after 158 days of litter exposure in the field may suggest that DOC primed litter SIR responses to increased nutrient availability. Apparently, the addition of cellulose had a different effect compared to litter inherent DOC. Cellulose decomposition requires particular enzymatic activities while DOC is a cocktail of various and easily accessible C-compounds that are likely used by a more diverse microbial community and may be also more quickly mineralized by opportunistic microorganisms. In that sense, litter inherent DOC may have similar effects like sugar fertilization [44]–[45], confirming the importance of the C quality in determining heterotrophic responses to C additions [74].

Nutrient fertilization has previously been shown to stimulate mass loss of litter from one tree species in a lowland Costa Rican rainforest [34] and of the original site-specific litter mixture from the fertilized plots in montane forests of Hawai'i [41] and in a lowland Panamanian rainforest [36]. Here we additionally highlighted that fertilization effects on decomposers depended on litter species-specific initial quality at our study site in an Amazonian rainforest. These results underline the importance of tree species-specific litter input to the forest floor for the understanding of how decomposers respond to changes in external N and P availability. Consequently, potential shifts in tree species composition and/or losses of tree species diversity in the Amazon [75], [76], global change induced changes in litter quality [27] and changes in anthropogenic nutrient inputs [9], [77] may interactively affect decomposer communities, litter decomposition and organic matter turnover.

### Fauna effect on heterotrophic microbial functioning

The contribution of fauna, especially that of macrofauna (*e.g.* millipedes, isopods, termites), to the decomposition process in tropical wet forests is disproportionately higher compared to forests ecosystems at higher latitudes [57]. The fauna impact on decomposition was shown to be influenced by litter stoichiometry in the rainforest of French Guiana [72] and the abundance and composition of soil fauna communities depended on soil C∶P stoichiometry in a tropical Costa-Rican rainforest [58]. Accordingly, changes in the relative availability of nutrients and/or of substrate C quality are likely to affect the composition and activity of fauna communities, with potential indirect effects on heterotrophic microbial functioning as well. In our third hypothesis, we expected that increased fauna activity with fertilization would decrease SIR rates as a result of increased predation on microorganisms by litter-feeding fauna.

In line with our hypothesis we observed that fauna increased and decreased the positive NP effect on litter mass loss and on litter SIR, respectively (Figure 2). The stronger net NP effect on litter mass loss in presence of fauna may suggest enhanced litter-feeding with a higher availability of nutrients. Alternatively, this fauna response may result from intensified detritivore foraging on litter that is more heavily colonized by microorganisms. This hypothesis is supported by the higher SIR rates measured in litter fertilized with NP in the absence of fauna. Higher detritivore feeding may then have reduced microbial biomass by direct consumption and indirect physical disruption of the microbial communities, possibly explaining the lower NP effect on SIR in presence of fauna.

### Are heterotrophic processes in the litter layer and underlying soil distinctively affected by fertilization?

We stated in our fourth hypothesis that nutrient fertilization stimulates the SIR rates more in litter than in the soil because of wider C∶nutrient stoichiometries in litter compared to soil. We actually observed an overall positive effect of N fertilization on litter SIR, but a negative N effect on soil SIR (Figure 1) that is broadly in agreement with our hypothesis. Such negative N fertilization effect was not associated with potential fertilizer induced changes in soil pH (*data not shown*). These contrasting effects of N fertilization in litter and soil are in line with Berg & Matzner's [2] reasoning of distinct effects of N fertilization on organic matter breakdown depending on the stage of decomposition. During the initial stage of decomposition when mostly soluble compounds and cellulose are broken down, N fertilization should have positive effects and during later stages of decomposition when more recalcitrant lignin-like compounds dominate the remaining organic matter, N fertilization is expected to have rather negative effects [2], [68]. Accordingly, Neff and coll. [78] showed that labile C fractions, present during the early stages of decomposition, are consumed more rapidly when N is added. Suppression of soil respiration in tropical forests following N fertilization has been repeatedly reported [37]–[39], but the mechanisms underlying this response are not yet clarified. Slower decomposition of organic matter *via* the decrease of oxidative enzyme production [38], [79], [80], decrease in labile C pools [39], inhibition of microbial biomass [37], [81], or changes in microbial community structure [82] have been proposed as potential mechanisms.

In contrast to N fertilization, P fertilization stimulated litter SIR (+20%) and soil SIR (+16%, Figure 1) in similar ways. A positive P effect on soil microbial activity was expected at our site with P-poor soils indicating P-deficient conditions [28]. Similarly, Cleveland and coll. [33] reported that P availability constrained the total respiratory CO_2_-flux in a Costa Rican tropical forest, and that P fertilization increased the proportion of added dissolved organic matter that was converted to CO_2_ [34]. Much of the positive P effect on soil SIR observed here was driven by the combined fertilization with P and C (Figure 2) resulting in an overall higher stimulation of soil SIR than when fertilized with P only. This positive interaction of a combined P and C fertilization was the only indication of a stimulating C effect on soil microbial activity as we initially hypothesized. The positive response in SIR may indicate that soil microorganisms are simultaneously limited by low soil P and by the access to labile C. Collectively, our data suggest that altered nutrient inputs in the studied Amazonian rainforest distinctly affect decomposer communities in the litter layer and the underlying soil with contrasting effects on organic matter turnover that is further modified by the quality of organic C sources.

### Conclusions

Taken together, our data show that increasing inputs of N and P, and in particular of both of them together can considerably change microbial activity and litter decomposition in a low fertile Amazonian rainforest. These effects are modified by soil fauna and depend on the quality of plant litter, especially on its quantities of labile C compounds and P. Moreover, soil and litter microorganisms are distinctly affected by increasing N inputs that may change relative C and nutrient fluxes between the litter layer and the soil. In a context of strong rise of N and P deposition predicted for tropical regions [9], [77], our results suggest important consequences for biogeochemical cycles in tropical forest ecosystems, and that simultaneous global change-induced shifts in the quality of leaf litter input could modulate these effects. However, in order to compare our data with previous fertilization experiments we used nutrient concentrations that exceed predictions for nutrient depositions in tropical rainforests. The effects of anthropogenic depositions might thus be lower than those observed here and studies utilizing more realistic levels of nutrients will be needed to estimate their true impact.

## Supporting Information

Table S1 **Mean values of litter mass loss, litter SIR, and soil SIR for each of the six different litter species and each individual fertilization treatment separated into fine and coarse mesh litterbags.**
(DOC)

Figure S1 **Litter SIR as a function of litter mass loss across all litter species and fertilization treatments but separated into coarse (grey circles) and fine (open circles) mesh litterbags.** Lines indicated fitted exponential (solid line) or linear (dashed line) regressions for the two fauna treatments separately.
(DOC)
