# Supplementary material for: Distinct Microbial Limitations in Litter and Underlying Soil Revealed by Carbon and Nutrient Fertilization in a Tropical Rainforest
Source: PLoS One. 2012 Dec 13;7(12):e49990. doi: 10.1371/journal.pone.0049990 (PMC3521737; doi:10.1371/journal.pone.0049990)
Supplement: Table S1 — Mean values of litter mass loss, litter SIR, and soil SIR for each of the six different litter species and each individual fertilization treatment separated into fine and coarse mesh litterbags. (DOC) [file pone.0049990.s001.doc]

**Table S1. Mean values of litter mass loss, litter SIR, and soil SIR for each of the six different litter species and each individual fertilization treatment separated into fine and coarse mesh litterbags.**

| Data | | *C. procera* | |  | *G. glabra* | |  | *H. courbaril* | |  | *P. insignis* | |  | *S. amara* | |  | *V. tomentosa* | |
| --- | --- | --- | --- | --- | --- | --- | --- | --- | --- | --- | --- | --- | --- | --- | --- | --- | --- | --- |
|  |  | fine | coarse |  | fine | coarse |  | fine | coarse |  | fine | coarse |  | fine | coarse |  | fine | coarse |
| Control | Mass loss (% DM) | 32.1 | 30.3 |  | 36.9 | 68.3 |  | 37.2 | 33.0 |  | 31.9 | 55.9 |  | 28.9 | 35.7 |  | 23.5 | 42.0 |
|  | SIR litter (µg g^-1^ h ^-1^) | 13.5 | 12.4 |  | 20.9 | 24.4 |  | 24.8 | 19.3 |  | 19.3 | 19.4 |  | 25.6 | 21.0 |  | 12.9 | 12.9 |
|  | SIR soil (µg g^-1^ h ^-1^) | 1.41 | 0.93 |  | 1.85 | 1.81 |  | 0.89 | 1.17 |  | 1.44 | 1.28 |  | 1.24 | 1.79 |  | 1.15 | 1.63 |
| C | Mass loss (% DM) | 28.5 | 19.0 |  | 38.9 | 55.0 |  | 32.9 | 30.1 |  | 34.1 | 53.6 |  | 30.7 | 31.8 |  | 26.2 | 31.7 |
|  | SIR litter (µg g^-1^ h ^-1^) | 15.6 | 18.2 |  | 23.0 | 23.6 |  | 26.1 | 16.8 |  | 20.8 | 20.7 |  | 31.3 | 22.2 |  | 11.8 | 13.9 |
|  | SIR soil (µg g^-1^ h ^-1^) | 1.21 | 1.34 |  | 1.45 | 1.32 |  | 1.34 | 1.11 |  | 1.33 | 1.19 |  | 1.28 | 1.40 |  | 1.18 | 1.27 |
| N | Mass loss (% DM) | 34.0 | 33.6 |  | 51.0 | 71.7 |  | 36.1 | 36.7 |  | 39.1 | 43.8 |  | 34.5 | 42.5 |  | 31.0 | 35.5 |
|  | SIR litter (µg g^-1^ h ^-1^) | 19.7 | 17.6 |  | 37.1 | 29.6 |  | 29.6 | 26.0 |  | 37.3 | 21.8 |  | 34.5 | 28.2 |  | 15.1 | 18.3 |
|  | SIR soil (µg g^-1^ h ^-1^) | 1.09 | 1.70 |  | 1.47 | 1.31 |  | 1.21 | 1.53 |  | 0.99 | 1.61 |  | 1.42 | 1.41 |  | 1.27 | 1.23 |
| P | Mass loss (% DM) | 31.4 | 27.9 |  | 40.0 | 71.9 |  | 36.7 | 34.9 |  | 41.9 | 75.0 |  | 35.8 | 35.1 |  | 25.5 | 35.4 |
|  | SIR litter (µg g^-1^ h ^-1^) | 17.1 | 12.5 |  | 33.1 | 21.1 |  | 23.6 | 18.7 |  | 30.2 | 14.5 |  | 27.5 | 19.6 |  | 13.0 | 14.4 |
|  | SIR soil (µg g^-1^ h ^-1^) | 1.24 | 1.57 |  | 1.20 | 1.31 |  | 1.84 | 1.69 |  | 1.57 | 1.50 |  | 1.36 | 1.56 |  | 1.62 | 2.05 |
| CN | Mass loss (% DM) | 29.1 | 35.8 |  | 40.9 | 70.4 |  | 39.6 | 26.4 |  | 38.0 | 60.7 |  | 28.4 | 30.8 |  | 28.4 | 48.8 |
|  | SIR litter (µg g^-1^ h ^-1^) | 17.1 | 21.4 |  | 41.1 | 20.5 |  | 33.0 | 30.8 |  | 24.1 | 21.1 |  | 37.6 | 31.1 |  | 17.9 | 19.3 |
|  | SIR soil (µg g^-1^ h ^-1^) | 1.24 | 0.70 |  | 1.09 | 1.06 |  | 1.37 | 1.00 |  | 1.15 | 1.18 |  | 1.29 | 1.21 |  | 1.20 | 1.01 |
| CP | Mass loss (% DM) | 33.8 | 35.4 |  | 43.1 | 76.3 |  | 42.5 | 24.6 |  | 41.4 | 85.5 |  | 33.0 | 33.4 |  | 25.3 | 46.9 |
|  | SIR litter (µg g^-1^ h ^-1^) | 15.5 | 18.4 |  | 29.1 | 21.2 |  | 27.9 | 22.3 |  | 38.2 | 39.6 |  | 32.8 | 31.1 |  | 17.3 | 23.5 |
|  | SIR soil (µg g^-1^ h ^-1^) | 2.12 | 1.37 |  | 1.78 | 1.36 |  | 1.88 | 1.72 |  | 2.23 | 1.55 |  | 1.98 | 2.06 |  | 1.65 | 2.05 |
| NP | Mass loss (% DM) | 33.9 | 44.5 |  | 45.3 | 80.7 |  | 40.2 | 37.2 |  | 44.0 | 89.5 |  | 37.3 | 43.1 |  | 33.8 | 57.2 |
|  | SIR litter (µg g^-1^ h ^-1^) | 24.7 | 22.8 |  | 55.0 | 45.4 |  | 34.5 | 32.3 |  | 41.3 | NA |  | 52.2 | 31.8 |  | 24.0 | 21.2 |
|  | SIR soil (µg g^-1^ h ^-1^) | 1.27 | 1.08 |  | 1.60 | 1.63 |  | 1.35 | 1.23 |  | 1.28 | 1.28 |  | 1.61 | 1.20 |  | 1.54 | 1.49 |
| CNP | Mass loss (% DM) | 36.5 | 52.8 |  | 45.9 | 90.8 |  | 35.5 | 28.1 |  | 45.9 | 87.2 |  | 35.3 | 35.1 |  | 35.8 | 52.5 |
|  | SIR litter (µg g^-1^ h ^-1^) | 23.7 | 28.6 |  | 51.1 | NA |  | 31.4 | 30.3 |  | 39.1 | 54.8 |  | 64.6 | 37.1 |  | 24.0 | 23.7 |
|  | SIR soil (µg g^-1^ h ^-1^) | 1.23 | 0.91 |  | 1.82 | 1.33 |  | 1.62 | 1.35 |  | 1.33 | 1.67 |  | 1.15 | 1.37 |  | 1.10 | 1.58 |
| +other nut. | Mass loss (% DM) | 27.1 | 27.0 |  | 36.9 | 61.3 |  | 36.3 | 24.2 |  | 34.4 | 54.5 |  | 28.4 | 28.0 |  | 29.3 | 30.0 |
|  | SIR litter (µg g^-1^ h ^-1^) | 10.9 | 16.1 |  | 25.9 | 29.7 |  | 24.2 | 20.9 |  | 19.5 | 21.7 |  | 28.9 | 23.5 |  | 15.0 | 17.6 |
|  | SIR soil (µg g^-1^ h ^-1^) | 0.88 | 1.06 |  | 0.97 | 1.08 |  | 1.13 | 1.33 |  | 1.17 | 0.93 |  | 0.93 | 0.78 |  | 0.93 | 1.31 |
