# Supplementary material for: Distinct Microbial Limitations in Litter and Underlying Soil Revealed by Carbon and Nutrient Fertilization in a Tropical Rainforest
Source: PLoS One. 2012 Dec 13;7(12):e49990. doi: 10.1371/journal.pone.0049990 (PMC3521737; doi:10.1371/journal.pone.0049990)
Supplement: Figure S1 — Litter SIR as a function of litter mass loss across all litter species and fertilization treatments but separated into coarse (grey circles) and fine (open circles) mesh litterbags. Lines indicated fitted exponential (solid line) or linear (dashed line) regressions for the two fauna treatments separately. (DOC) [file pone.0049990.s002.doc]

**Figure S1. Litter SIR as a function of litter mass loss across all litter species and fertilization treatments but separated into coarse (grey circles) and fine (open circles) mesh litterbags. Lines indicated fitted exponential (solid line) or linear (dashed line) regressions for the two fauna treatments separately.**
